# Monitoring cheese ripening by single-sided nuclear magnetic resonance

**DOI:** 10.3168/jds.2022-22458

**Published:** 2023-03

**Authors:** Y. Kharbanda, S. Mailhiot, O. Mankinen, M. Urbańczyk, V.-V. Telkki

**Affiliations:** 1 NMR Research Unit, University of Oulu, 90570 Oulu, Finland; 2 Institute of Physical Chemistry, Polish Academy of Sciences, 01-224 Warsaw, Poland

**Keywords:** cheese ripening, single-sided nuclear magnetic resonance (NMR), Laplace NMR, spatial profiling

## Abstract

The noninvasive, longitudinal study of products and food processing is of interest for the dairy industry. Here, we demonstrated that single-sided nuclear magnetic resonance (NMR) can be used for noninvasive monitoring of the cheese ripening process. The maturation of soft-ripened Camembert-like molded cheese samples was monitored for 20 d measuring 1-dimensional and 2-dimensional NMR relaxation and diffusion data at various depths, ranging from the hard surface layer to the soft center. Gelation and gel shrinkage were observed throughout ripening, and a complete loss of free water signal was observed at the cheese rind. Transversal (*T*_2_) relaxation distributions include 3 components that evolve with ripening time and position, corresponding to water inside the casein gel network, water trapped in casein, and fat. Two-dimensional *T*_1_-*T*_2_ relaxation experiments provided enhanced resolution of the 3 components, allowing quantification of the relative proportions of each phase. Furthermore, diffusion (*D*)-*T*_2_ relaxation correlation experiments revealed the bimodal size distribution of fat globules. The study demonstrated that single-sided NMR can provide spatially resolved signal intensity, relaxation, and diffusion parameters that reflect structural changes during the ripening process and can be exploited to understand and monitor the ripening of cheeses.

## INTRODUCTION

Cheese is a dairy product derived from milk that can have a range of flavors, textures, and shapes depending on source materials and production conditions (Fox et al., 2004). Cheese production for acid-set or rennet cheeses can be divided into 3 steps: milk processing, gelation, and ripening. During milk processing, lactose is fermented into lactic acid (Braun et al., 2019). Gelation occurs after the addition of acid or rennet to the milk, which causes the formation of casein aggregates or cheese curd (Kapoor and Metzger, 2008; Gamlath et al., 2020). Ripening is the most time-consuming part of production and is defined as the storage of cheese over time and under specific conditions. The ripening process is a series of physical, chemical, and biological changes that take place over time (Farkye, 2014).

**Figure 1:**
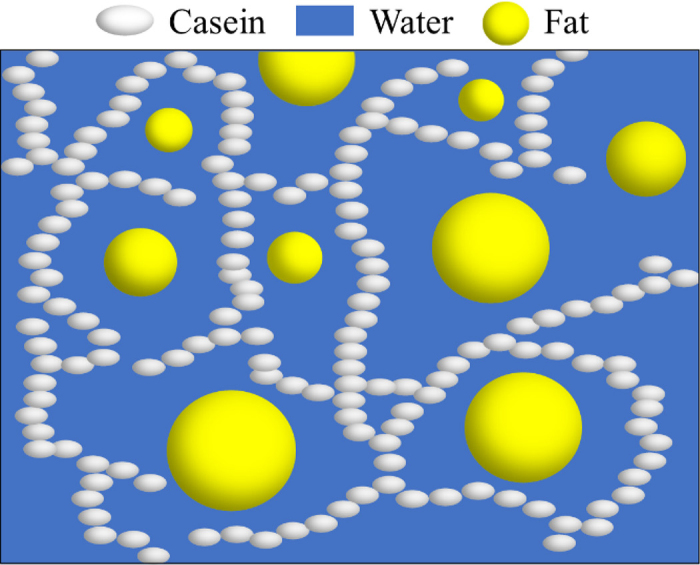
A schematic diagram of the cheese gel network. Cheese is an emulsion of liquid fat globules in a fluid phase composed of water and casein proteins. The casein proteins form a continuous pore network.

During cheese production, a cheese gel network is formed (Figure 1). Cheese is an emulsion of milk fat in a fluid phase. The fat consists primarily of triglycerides in spherical globules. At room temperature, a portion of the fat is liquid (Chaland et al., 2000). The fluid phase of bovine milk is composed of water, casein, and lactose (McGee, 2007). In milk, casein proteins form micelles, whereas in cheese, casein proteins form a continuous porous network. During cheese ripening, this porous network of water, liquid fat, and casein evolves based on factors such as milk quality, milk type, milk enzymes, rennet or substitute, starter bacteria, mold, and temperature (Fox et al., 2004). It is crucial to understand how these factors affect cheese quality, flavor, and texture (Fox et al., 2004).

Nuclear magnetic resonance (**NMR**) techniques have been widely exploited in food science (Hills, 1994; Webb et al., 2001; Mariette, 2018). Nuclear magnetic resonance is a promising technique because it is noninvasive, sensitive to variables of interest such as water and fat content (van den Enden et al., 1982; Gribnau, 1992; Mariette, 2009), and, in the case of low-field NMR techniques, affordable. In low-field NMR, relaxometry (Le Dean et al., 2004) and diffusometry (Watanabe and Fukuoka, 1992) can be used for characterization without relying on the chemical shift resolution of traditional NMR experiments (Hills, 2006). Transversal (***T*_2_**) relaxation is sensitive to the rotational mobility of molecules, which is affected by composition and chemical structure, as well as molecular exchange (Mariette, 2018). *T*_2_ experiments are fast (typical experiment time is on the order of minutes) and easy to automate. Diffusion experiments provide direct information about Brownian motion of molecules, reflecting molecular and aggregate sizes. Multidimensional correlation experiments improve the resolution and information content of relaxometry and diffusometry (Hürlimann et al., 2006; Song, 2009). For example, *T*_1_-*T*_2_ experiments enable correlation of longitudinal (***T*_1_**) and transverse relaxation times, whereas *D*-*T*_2_ experiments correlate the molecular diffusion coefficient (***D***) with *T*_2_ relaxation time, allowing, for example, the identification of water and liquid fat components (Hürlimann et al., 2006; Song, 2009).

**Figure 2:**
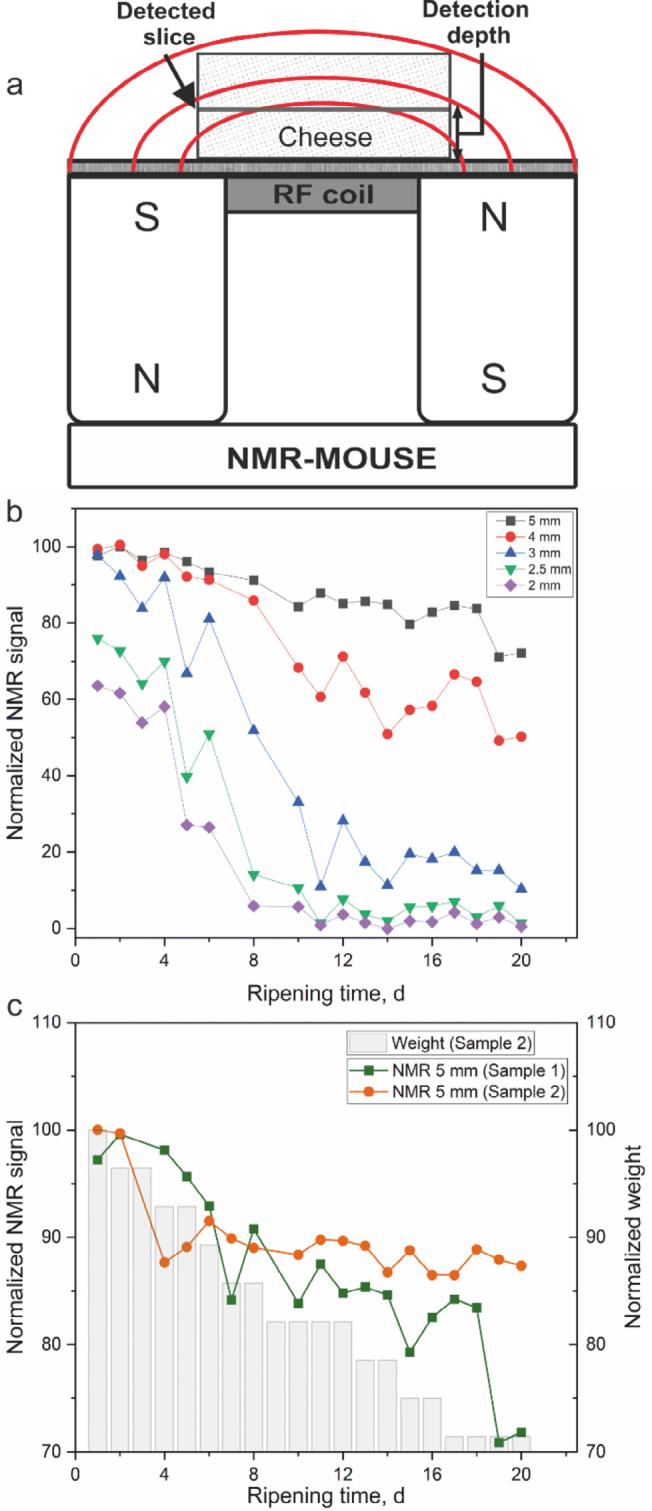
(a) Schematic diagram of the single-sided nuclear magnetic resonance (NMR)-Mouse used for monitoring cheese ripening. (b) ^1^H signal intensity of sample 1 as a function of ripening time at different depths (2 to 5 mm). The intensity is the amplitude of the fifth echo of the Carr-Purcell-Meiboom-Gill (CPMG) data, corresponding to total echo time of 1 ms. (c) Normalized NMR signal intensities of samples 1 and 2 and the normalized weight of sample 2 as a function of ripening time at a depth of 5 mm. In the normalization, the maximum NMR signal and weight was set to 100. S = south pole of the magnet, N = north pole of the magnet, RF coil = radiofrequency coil.

One type of low-field NMR device is the single-sided NMR device (Blümich et al., 1998, 2002; Blümich, 2016). A single-sided NMR device is one in which the magnetic field extends from the surface of the magnet. The field decreases with increasing distance from the surface, and signal is observed from a thin layer in which the Larmor frequency of spins matches with the resonant frequency of the radiofrequency coil. With single-sided NMR devices, the sample is placed on top of the magnet instead of inside the magnet, such that there is no sample size requirement or change in condition. Using such a set-up, the depth-dependent evolution of a sample can be studied (Blümich et al., 1998; Figure 2). The present work used single-sided NMR for a longitudinal, depth-dependent study of soft-ripened molded Camembert-like cheeses. Such depth-dependent information is beneficial when the maturation process differs significantly depending on the region of product. In this study, we monitored the 20-d ripening process longitudinally by measuring ^1^H signal intensity, *T*_2_, *T*_1_-*T*_2_, and *D*-*T*_2_ data daily at the surface and inside the soft center of the cheeses.

## MATERIALS AND METHODS

### Cheese Preparation

Each cheese was made from 400 mL of pasteurized milk containing 3.2% fat (Valio Oy), 2 mg of calcium chloride (Biochem s.r.l.), 2 mg of bacteria starter culture (Biochem s.r.l.), 2 mg of mold (Biochem s.r.l.; lyophilized *Penicillium candidum*), and 500 µL of rennet (Åströmin Jälk. Oy). The starter culture was a lyophilized mixture of *Lactococcus lactis* ssp. *lactis*, *L. lactis* ssp. *cremoris, L. lactis* ssp. *lactis* biovar *diacetylactis*, and *Streptococcus salivarius* ssp. *thermophilus*. The milk was warmed for 15 min at 30°C, and then calcium chloride was added to the milk. The mixture was stirred for 5 min with a spatula. Then, the bacteria and mold were added, and the mixture was left to stand for 15 min at 30°C. Finally, rennet was added and the mixture was left for 30 min at room temperature. The mixture was then filtered using a custom-made colander made from a plastic box with small perforations in the bottom. The remaining solid mass in the box was pressed for 12 h to drain the excess whey. Finally, the cheese was cut into 5 × 5 cm sections and salted with non-iodized salt. The samples were cured for 20 d in a room at 21°C and 15% humidity. Ripening measurements were performed on 2 different samples. Weight measurements were taken every day on one of the samples.

### NMR Experiments

The NMR experiments were performed using a Magritek Profile NMR-Mouse PM 25 with an operating ^1^H resonance frequency of 13.29 MHz and 7.28 Tm^−1^ gradient, Kea 2 spectrometer, and Prospa software (Magritek). The cheese sample was set on the top of the NMR-Mouse (Figure 2a), and *T*_2_ relaxation and *T*_1_-*T*_2_ and *D*-*T*_2_ correlation experiments were performed at 5 positions: 5, 4, 3, 2.5, and 2 mm from the surface of the device. The 5-mm position corresponds to the soft center of the cheese sample and the 2-mm position corresponds to the outer layer (skin) of the cheese. Experiments were performed once a day for 20 d at room temperature.

**Figure 3:**
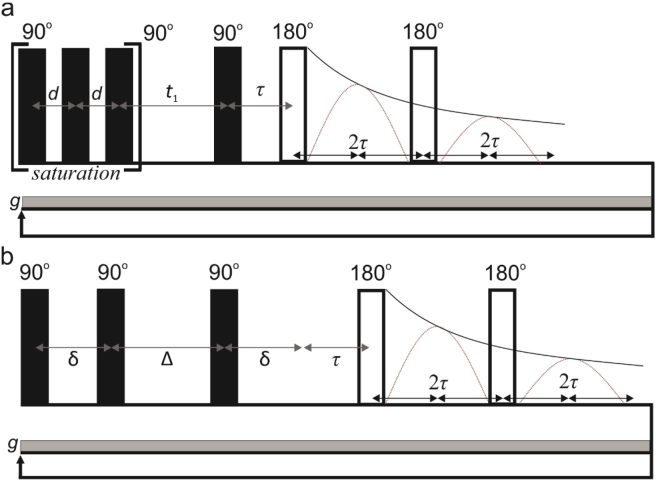
Pulse sequences for (a) *T*_1_-*T*_2_ and (b) *D*-*T*_2_ correlation experiments. Δ= diffusion encoding time, δ = time between two 90° pulses, τ = echo time.

The *T*_2_ relaxation data were measured using a Carr-Purcell-Meiboom-Gill (**CPMG**) sequence (Meiboom and Gill, 1958) with a 200-µs echo time (2τ), 512 echoes (*n*), 2.5-s repetition time, and 128 scans; the experiment time was 5.8 min. The *T*_1_-*T*_2_ data (Peemoeller et al., 1981) were measured with a pulse sequence consisting of a saturation recovery (Becker et al., 1980) block followed by a CPMG sequence in which data were recorded at the center of every echo (Figure 3a). Saturation was achieved using seven 90° radiofrequency pulses with an inter-pulse delay between 100 and 750 µs. The *T*_1_ recovery time (*t*_1_) was varied from 1 to 2,500 ms in 32 log spaced steps. A 200-µs echo time (2τ), 512 echoes (*n*), 32 scans, and 2.5-s repetition time were used. The experiment time was 44 min.

The signal (*S*) observed in the *T*_1_-*T*_2_ experiment is described as follows:

[1] $$S\left(t_{1},t_{2}\right)=\int \int P\left(T_{1},T_{2}\right)\left[1-exp\left(-\frac{t_{1}}{T_{1}}\right)\right]exp\left(-\frac{2n\tau }{T_{2}}\right)dT_{1}dT_{2},$$

where *P*(*T*_1_,*T*_2_) is the *T*_1_-*T*_2_ correlation probability distribution.

The *D*-*T*_2_ data (Hürlimann and Venkataramanan, 2002) were measured with a sequence including a stimulated echo sequence (Tanner, 1970) followed by a CPMG sequence in which data were recorded at the center of every echo (Figure 3b). The diffusion encoding was achieved by exponentially increasing the time between two 90° pulses (δ) from 0.1 to 5 ms with 32 steps. The diffusion delay (Δ) was 5 ms. The number of echoes in the CPMG was 512, the echo time was 200 µs, the number of scans was 32, and the repetition time was 2.5 s. The experiment time was 43 min. The signal (*S*) observed in the *D*-*T*_2_ is given as follows:

[2] $$\begin{array}{c} S\left(\delta ,t\right)=\int \int P\left(D,T_{2}\right)\\ exp\left(-\gamma^{2}g^{2}D\delta^{2}D\left(\Delta -\frac{\delta }{3}\right)\right)exp\left(-\frac{2n\tau }{T_{2}}\right)dDdT_{2}, \end{array}$$

where γ is the gyromagnetic, *g* is the gradient strength, δ is the time between 90° pulses, Δ is the diffusion encoding time, and *P*(*D*, *T*_2_) is the *D*-*T*_2_ correlation probability distribution.

### Data Analysis

^1^H signal intensity was defined as the signal at the fifth echo of the CPMG sequence corresponding to *S*(*t* = 1 ms). The *T*_2_ probability distribution, *P*(*T*_2_), was calculated from CPMG data using a 1-dimensional (**1D**) inverse Laplace transform (**ILT**) with ITAMeD implementation (Urbańczyk et al., 2013). The *P*(*T*_1_, *T*_2_) and *P*(*D*, *T*_2_) distributions were calculated using the 2-dimensional (**2D**) ILT (Venkataramanan et al., 2002; Song, 2009; Granwehr and Roberts, 2012) with ITAMeD implementation (Urbańczyk et al., 2013). Complete sets of *D*-*T*_2_ and *T*_1_-*T*_2_ maps with all depths are shown in the supplemental data (Supplemental Figures S1 and S2, respectively; https://doi.org/10.5281/zenodo.7197359; Kharbanda et al., 2022). The center and area of the 2D peak was defined by first fitting the 2D Gaussian function to each peak. The center of the peak was extracted directly from the model, and the area was calculated by integrating the fitted Gaussian function.

The results were further compared with another 2D ILT algorithm, Upen2DTool (Bortolotti et al., 2019) based on the uniform penalty principle, to confirm the validity of the results (shown in Supplemental Figure S3; https://doi.org/10.5281/zenodo.7197359; Kharbanda et al., 2022).

## RESULTS AND DISCUSSION

Cheese ripening was studied for the first time longitudinally in 2 soft-ripened molded Camembert-like cheese samples (called samples 1 and 2), at several depths, without alteration to the samples. These measurements were made possible by using single-sided NMR because it can measure samples of any size. Based on previous research, the obtained *D*, *T*_1_, and *T*_2_ peaks were assigned to water, fat, and a protein-associated peak (Wolter and Krus, 2006; Capitani et al., 2017). There is a long history of using NMR relaxation to identify these peaks, starting in 1958 when the first NMR study of milk was published, in which the authors identified 3 distinct components present in milk, water, lactose, and fat (Odeblad and Westin, 1958).

In the study of cheese ripening, the effect of gel shrinkage and water evaporation is first considered using ^1^H signal intensity. ^1^H NMR signal intensity of sample 1 evolved during maturation as a function of ripening time and distance from the surface (Figure 2b). As the *T*_2_ relaxation time of nonexchangeable protons of macromolecules (20–30 µs; Bouchoux et al., 2012) is much shorter than the 200-µs echo time, the proton signal intensity reflects the total amount of mobile water, fat, and exchangeable protons. In the soft center 5 mm from the magnet surface, the intensity decreased continuously over the 20-d period, predominantly due to water evaporation, and the total signal decrease was about 30%. In the outermost layer, at a depth of 2 mm from the magnet surface, the signal intensity at the beginning of the ripening process was about 36% less than in the center layer, and the signal vanished on d 8 due to formation of the dry skin layer. The drying rate and overall signal depression decreased with increasing depth. The loss of signal intensity is a strong indicator, perhaps the most unambiguous indication of cheese ripening in cheeses that form a rind. When the signal is lost at the surface of the cheese, it implies that the rind is forming, which is a critical step in the ripening of this type of cheese. Additionally, the depth-dependent measurements of signal showed development of a thick rind. The total decrease in NMR signal intensity (13%) in the soft center (depth 5 mm) of sample 2 during the ripening period was smaller than that of sample 1 (30%; Figure 2c). We interpret this to reflect the slower ripening of the former sample; after ripening, sample 2 was softer than sample 1, supporting this interpretation. Both the normalized NMR signal and normalized weight of sample 3 decreased during the ripening process (Figure 2c), similarly and qualitatively reflecting the drying of cheese. However, the former decrease was smaller because the NMR signal reflects spin density of mobile protons, whereas mass reflects the overall number of molecules in a sample, which depends on both density and volume of the sample.

**Figure 4:**
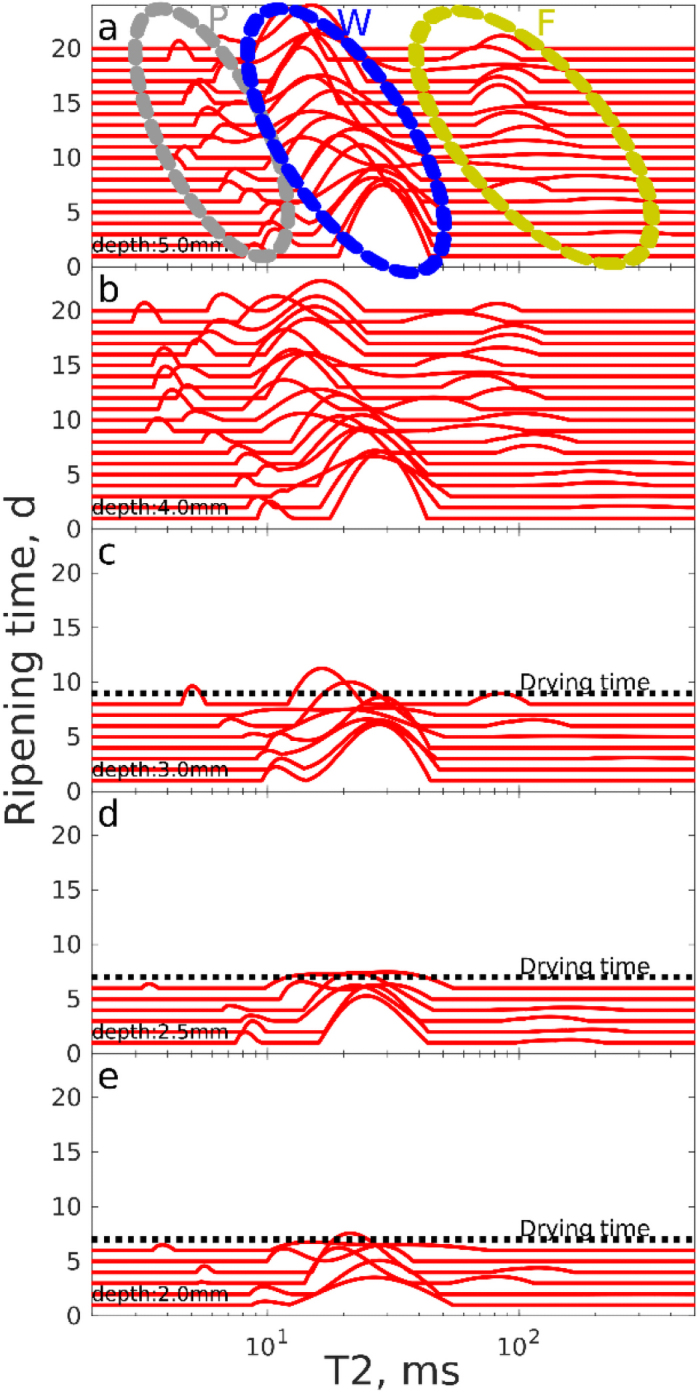
^1^H *T*_2_ distributions of sample 1 as a function of ripening time. P represents water molecules trapped in casein protein, W indicates the signal of water inside the mesh of the casein gel network, and F represents the fat signal. The drying times of different layers are illustrated by black dashed lines. Drying time was defined as a signal to noise ratio <30 and reflects the rate at which different depths of the cheese rind ripened.

To gain information about the evolution of the heterogeneous cheese structure (Baudrit et al., 2010; Lamichhane et al., 2018; Figure 1), the NMR parameters *T*_2_, *T*_1_, and *D* are used. *T*_2_ is affected by the porous structure and exchange processes as a result of water–macromolecule interactions (Hills et al., 1993). In this study, *T*_2_ relaxation was measured as a function of cheese ripening time and showed 3 components (Figure 4). In the soft center of the cheese 5 mm from the magnet surface, the *T*_2_ distributions included a dominant peak around *T*_2_ = 30 ms and 2 smaller peaks around *T*_2_ = 10 and 100 ms. The *T*_2_ relaxation times decreased with increasing ripening time. This is interpreted to be a consequence of ripening, gel shrinkage, and water evaporation, which increase interactions between the remaining water molecules and proteins, leading to shortened *T*_2_, for example, due to enhanced chemical exchange. The examination of all 3 peaks has been reported in several studies (Markley et al., 1971; Brosio and Barbieri, 1996; Le Dean et al., 2004). The shortest relaxation time peak, peak P (Figure 4), was ascribed to water trapped within casein, as its *T*_2_ relaxation time (about 10 ms) is close to the *T*_2_ values of the 2 casein-associated water peaks (7.2 and 16.2 ms) observed by Gianferri et al. (2007) for mozzarella cheese. Observation of only a single casein-associated peak may be a consequence of different structure of the Camembert-like cheese, accelerated exchange between the 2 sites due to the higher measurement temperature in our studies, lower resolution in our experiment, or an effect of diffusion in the constant gradient field on the apparent observer *T*_2_ (see below). The dominant intermediate *T*_2_ peak, labeled W, was attributed to highly mobile water present in the gel structure of the cheese. The high mobility was confirmed by the *D*-*T*_2_ measurements described below. The *T*_2_ of the water peak (about 30 ms) was much shorter than that of corresponding peak (488 ms) observed in mozzarella cheese by Gianferri et al. (2007) and other previous studies (Hürlimann et al., 2006; Tidona et al., 2021; Alinovi et al., 2022). This is an effect of fast diffusion of free water in the strong constant field gradient of the single-sided instrument used in the current study. In the presence of constant gradient *g*, the observed apparent relaxation time *T*_2app_ in the CPMG experiment can be calculated using the following equation (Carr and Purcell, 1954):

[3] $$\frac{1}{T_{2app}}=\frac{1}{T_{2}}+\frac{\gamma^{2}\tau^{2}g^{2}D}{3}.$$

If we assume that “true” *T*_2_ relaxation time of water without diffusion effect is 488 ms, as determined by Gianferri et al. (2007), and use values relevant for our experiments (τ = 100 μs, *g* = 7.28 T/m, *D* = 2⋅10^−9^ m^2^/s), Eq. [3] gives *T*_2app_ = 36 ms, which is in good agreement with the *T*_2_ of peak W observed in our experiments. The peak with the longest relaxation time was assigned to signal from protons in the lipid phase, F (Schlesser et al., 1992; Gianferri et al., 2007). These peaks showed the same *T*_2_ relaxation time in the center of the cheese and at the skin before formation of the skin layer. We noted that the effect of diffusion on the apparent *T*_2_ was smaller for the fat- and casein-associated water signals due to their shorter “true” *T*_2_ and slower diffusion (see Eq. [3]). During the maturation process, *T*_2_ values of all peaks of sample 1 decreased significantly more than those of sample 2 [compare Figure 4 with Supplemental Figure S4 (https://doi.org/10.5281/zenodo.7197359; Kharbanda et al., 2022)] due to the slower ripening of sample 2. Consequently, *T*_2_ is another sensitive parameter reflecting the degree of maturation in addition to the NMR signal strength (see above).

This characterization of a single parameter *T*_2_ can be prone to misinterpretation. In the early 2000s, Métais and Mariette (2003) performed a quantitative analysis of fat and water in fatty protein concentrate and showed that *T*_2_ of the fat proton covers a wide range, which is overlaid with the water proton relaxation time. Moreover, dairy products consist not only of fat and water, but also contain protein and sugar and, depending on the preparation, the system may become more complex. To address this issue, 2D NMR correlation experiments can be used, such as *T*_1_-*T*_2_ and *D*-*T*_2_ methods. In correlation experiments, the data are resolved using 2 correlated variables, which can improve peak separation and identification. In *T*_1_-*T*_2_ correlation experiments, the *T*_1_:*T*_2_ ratio can be identified for each component, which reflects mobility, interactions, and the state of molecules (Song et al., 2002). In free liquids, *T*_1_ = *T*_2_, whereas in solid-like materials, *T*_1_ is much longer than *T*_2_. On the other hand, as described above, the strong, constant gradient of the NMR-Mouse coupled with molecular diffusion accelerates the signal decay during the CPMG loop (Blümich et al., 2002), resulting in measurement of a faster, diffusion-weighted *T*_2_ relaxation rate and subsequently higher *T*_1_ or *T*_2_ relaxation rate for quickly diffusing liquids but not slowly diffusing liquids such as fat.

**Figure 5:**
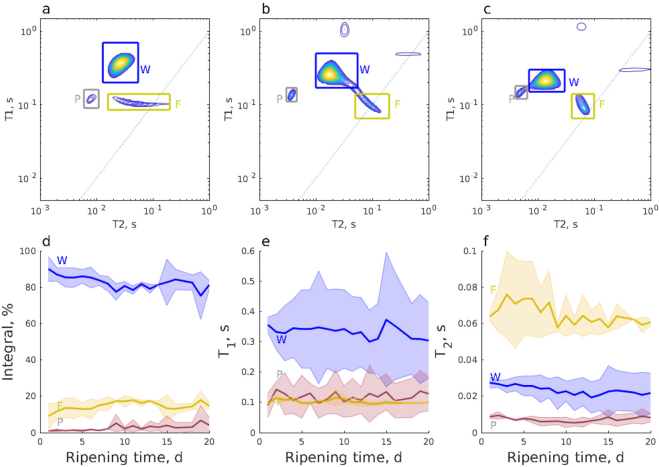
*T*_1_-*T*_2_ maps of sample 1 measured from the center layer at the depth of 5 mm at (a) d 1, (b) d 10, and (c) d 20. The blue dashed lines represent *T*_1_ = *T*_2_. (d) Integrals of the signals as a function of the ripening time; and (e) *T*_1_ and (f) *T*_2_ relaxation rates of the water, protein, and fat peaks. The shaded areas represent the standard deviations of the measurements of samples 1 and 2, and the values presented in panels (d)–(f) represent the mean values of the performed measurements. P represents water molecules trapped in casein protein, W indicates the signal of water inside the mesh of the casein gel network, and F represents the fat signal.

The *T*_1_-*T*_2_ correlation map of sample 1 measured on d 1 of ripening (Figure 5a) included 3 signals. One signal (W), around *T*_1_ = 320 ms and *T*_2_ = 60 ms, was interpreted to arise from water. The second signal (P) was interpreted to be water trapped in casein, with *T*_1_ = 160 ms and *T*_2_ = 2.4 ms. The *T*_1_:*T*_2_ ratio is high, about 65, because chemical exchange is a particularly efficient relaxation mechanism for *T*_2_ relaxation (Hills et al., 1990; Józef and Lena, 2017). The third signal with *T*_1_ = 100 ms and *T*_2_ = 70 ms was hypothesized to arise from liquid fat (Song, 2009). The fat peak was characterized by liquid-like behavior with a *T*_1_:*T*_2_ ratio ≈ 1; therefore, we interpret that fat is predominantly in liquid form in the cheese. The integrals of the peaks in the *T*_1_-*T*_2_ maps (Figure 5d) indicate that the relative amount of water decreased by about 20% during the 20-d ripening period, whereas the protein and fat peaks increased as a function of ripening time. The peak assignment used here is in agreement with previous studies (Hills et al., 2004; Hürlimann et al., 2006). In addition to the 3 peaks discussed above, the *T*_1_-*T*_2_ maps include some additional peaks, which were interpreted to be artifacts arising from experimental noise. Such artifactual peaks are unavoidable due to the numerically unstable nature of ILT (Song et al., 2002; Telkki, 2018). The peaks were identified to be artifacts because their amplitude was small, they were not reproducible, and some of them were not physically acceptable due to having a longer *T*_2_ than *T*_1_.

**Figure 6:**
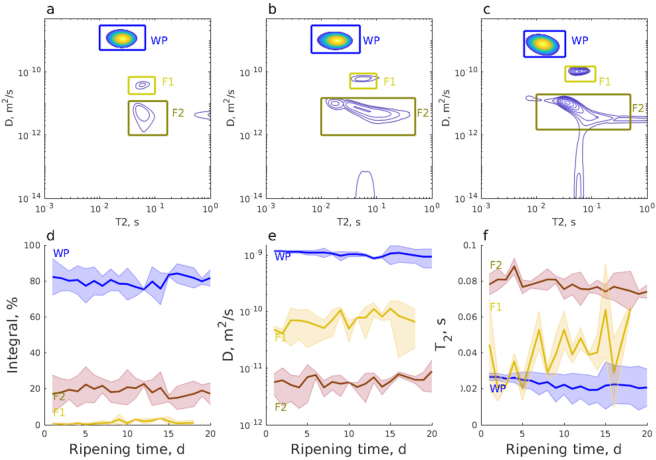
*D*-*T*_2_ maps of sample 1 taken from the center layer at the depth of 5 mm at (a) d 1, (b) d 10, and (c) d 20; and (d) integral, (e) diffusion coefficient (D), and (f) *T*_2_ relaxation times of the water and fat peaks. The shaded areas represent the standard deviation of the measurements of samples 1 and 2, and the values presented in panels (d)–(f) represent the mean values of the performed measurements. P represents water molecules trapped in casein protein, W indicates the signal of water inside the mesh of the casein gel network, and F represents the fat signal.

Another NMR method that can be used to understand the ripening process is *D*-*T*_2_; it is well suited for quantifying the fat and moisture content of food products (Watanabe and Fukuoka, 1992). The diffusion coefficient of free water (*D*) = 2 × 10^−9^ m^2^·s^−1^ at room temperature; *D* decreases as molecular size increases, such as for protein or fat, and when mobility is restricted by the structure (Brownstein and Tarr, 1979). During ripening, 3 peaks were observed in *D*-*T*_2_ maps (Figure 6). The dominant peak with the highest diffusion coefficient (around 10^−9^ m^2^·s^−1^, labeled “WP”) was interpreted to arise from water. We anticipate that the signal of water trapped in casein was fused with the water signal in the *D*-*T*_2_ maps due to their similar *D* and *T*_2_ values; the exchange between the casein-trapped water and gel pore water pools are expected to be relatively fast in the diffusion delay time scale, leading to similar *D* values for both sites. The *D* associated with the WP peak decreased as ripening progressed due to more restricted diffusion in the casein gel network. The 2 peaks with significantly lower diffusion coefficients (<10^−10^ m^2^·s^−1^, labeled F1; and <10^−11^ m^2^·s^−1^, labeled F2) are interpreted to originate fat globules, which are known to have *D* values around that range (Hürlimann et al., 2006). The observation of 2 fat peaks indicates 2 distinct fat globule sizes The differentiation of 2 types of fat globules confirms a previous confocal laser scanning microscopy study of Camembert-type cheese (Lopez, 2005). We expect that diffusion of fat molecules was restricted by the size of the fat globules. The mean square displacements *z* = (2Δ*D*)^1/2^ corresponding to *D* = 10^−10^ and 10^−11^ m^2^·s^−1^ (Δ = 5 ms) were 1 and 0.3 μm, respectively. Therefore, we interpreted that the lower diffusion coefficient peak F1 corresponded to smaller fat globules with the size <1 μm and the F2 peak arose from larger, >1 μm, fat globules. Similar size ranges have been observed by microscopy (Lopez, 2005). The relative intensity of the fat signal increased with ripening time, similarly to that of the *T*_1_-*T*_2_ experiments. The *D* values of both fat peaks remained quite constant over the 20-d ripening period, indicating that the fat globule sizes were stable (Lopez, 2005).

## CONCLUSIONS

In this work, we present a longitudinal study of cheese ripening using single-sided NMR. By studying ripening at the surface and inside the cheeses, the evolution of cheese gel structure was studied by following water signals associated with aqueous water, liquid fat, and casein protein. The skin formation was visible in the outermost layers (2–3 mm) and could be differentiated from the soft-ripening process in the central layers (4–5 mm) using ^1^H signal intensity. The overall decrease of signal in the central layer was interpreted to reflect the degree of ripening. Different components of cheeses were identified using 1D *T*_2_ relaxation and 2D *T*_1_-*T*_2_ and *D*-*T*_2_ correlation measurements. By monitoring the ripening process, the mobile water loss and gel shrinkage were observed, whereas the proportion of casein-associated water and fat increased. Furthermore, information was gained about the fluidity, sizes and stability of fat globules with 2 distinct sizes during ripening. Overall, this study shows the potential for single-sided NMR to be valuable tool for studying cheese ripening noninvasively.

## References

[bib1] Alinovi M., Tidona F., Monti L., Francolino S., Brusa G., Ghiglietti R., Locci F., Giraffa G. (2022). Physicochemical and rheological characteristics of Crescenza cheese made with 40% of recombined milk during manufacture and storage. Int. J. Dairy Technol..

[bib2] Baudrit C., Sicard M., Wuillemin P.H., Perrot N. (2010). Towards a global modelling of the Camembert-type cheese ripening process by coupling heterogeneous knowledge with dynamic Bayesian networks. J. Food Eng..

[bib3] Becker E.D., Ferretti J.A., Gupta R.K., Weiss G.H. (1980). The choice of optimal parameters for measurement of spin-lattice relaxation times. II. Comparison of saturation recovery, inversion recovery, and fast inversion recovery experiments. J. Magn. Reson..

[bib4] Blümich B. (2016).

[bib5] Blümich B., Anferov V., Anferova S., Klein M., Fechete R., Adams M., Casanova F. (2002). Simple NMR-MOUSE with a bar magnet. Concepts Magn. Reson..

[bib6] Blümich B., Blümler P., Eidmann G., Guthausen A., Haken R., Schmitz U., Saito K., Zimmer G. (1998). The NMR-mouse: Construction, excitation, and applications. Magn. Reson. Imaging.

[bib7] Bortolotti V., Brizi L., Fantazzini P., Landi G., Zama F. (2019). Upen2DTool: A uniform PENalty Matlab tool for inversion of 2D NMR relaxation data. SoftwareX.

[bib8] Bouchoux A., Schorr D., Daffé A., Cambert M., Gésan-Guiziou G., Mariette F. (2012). Molecular mobility in dense protein systems: An investigation through 1 H NMR relaxometry and diffusometry. J. Phys. Chem. B.

[bib9] Braun K., Hanewald A., Vilgis T.A. (2019). Milk emulsions: Structure and stability. Foods.

[bib10] Brosio E., Barbieri R. (1996). Nuclear magnetic resonance in the analysis of dairy products. Rev. Anal. Chem..

[bib11] Brownstein K.R., Tarr C.E. (1979). Importance of classical diffusion in NMR studies of water in biological cells. Phys. Rev. A Gen. Phys..

[bib12] Capitani D., Sobolev A.P., Di Tullio V., Mannina L., Proietti N. (2017). Portable NMR in food analysis. Chem. Biol. Technol. Agric..

[bib13] Carr H.Y., Purcell E.M. (1954). Effects of diffusion on free precession in nuclear magnetic resonance experiments. Phys. Rev..

[bib14] Chaland B., Mariette F., Marchal P., De Certaines J. (2000). 1H nuclear magnetic resonance relaxometric characterization of fat and water states in soft and hard cheese. J. Dairy Res..

[bib15] Farkye N.Y. (2014).

[bib16] Fox P., McSweeney P., Cogan T., Guinee T. (2004).

[bib17] Gamlath C.J., Leong T.S.H., Ashokkumar M., Martin G.J.O. (2020). Incorporating whey protein aggregates produced with heat and ultrasound treatment into rennet gels and model non-fat cheese systems. Food Hydrocoll..

[bib18] Gianferri R., Maioli M., Delfini M., Brosio E. (2007). A low-resolution and high-resolution nuclear magnetic resonance integrated approach to investigate the physical structure and metabolic profile of Mozzarella di Bufala Campana cheese. Int. Dairy J..

[bib19] Granwehr J., Roberts P.J. (2012). Inverse Laplace transform of multidimensional relaxation data without non-negativity constraint. J. Chem. Theory Comput..

[bib20] Gribnau M.C.M. (1992). Determination of solid/liquid ratios of fats and oils by low-resolution pulsed NMR. Trends Food Sci. Technol..

[bib21] Hills B., Benamira S., Marigheto N., Wright K. (2004). T1–T2 correlation analysis of complex foods. Appl. Magn. Reson..

[bib22] Hills B.P. (1994).

[bib23] Hills B.P. (2006).

[bib24] Hills B.P., Belton P.S., Quantin V.M. (1993). Water proton relaxation in heterogeneous systems. Mol. Phys..

[bib25] Hills B.P., Takacs S.F., Belton P.S. (1990). A new interpretation of proton NMR relaxation time measurements of water in food. Food Chem..

[bib26] Hürlimann M.D., Burcaw L., Song Y.Q. (2006). Quantitative characterization of food products by two-dimensional D-T2 and T1–T2 distribution functions in a static gradient. J. Colloid Interface Sci..

[bib27] Hürlimann M.D., Venkataramanan L. (2002). Quantitative measurement of two-dimensional distribution functions of diffusion and relaxation in grossly inhomogeneous fields. J. Magn. Reson..

[bib28] Józef K., Lena M. (2017).

[bib29] Kapoor R., Metzger L.E. (2008). Process cheese: Scientific and technological aspects—A review. Compr. Rev. Food Sci. Food Saf..

[bib30] Kharbanda Y., Mailhiot S., Mankinen O., Urbańczyk M., Telkki V.-V. (2022). Supplementary information for: Monitoring cheese ripening by single-sided NMR. Zenodo.

[bib31] Lamichhane P., Kelly A.L., Sheehan J.J. (2018). Symposium review: Structure-function relationships in cheese. J. Dairy Sci..

[bib32] Le Dean A., Mariette F., Marin M. (2004). ^1^H nuclear magnetic resonance relaxometry study of water state in milk protein mixtures. J. Agric. Food Chem..

[bib33] Lopez C. (2005). Focus on the supramolecular structure of milk fat in dairy products. Reprod. Nutr. Dev..

[bib34] Mariette F. (2009). Investigations of food colloids by NMR and MRI. Curr. Opin. Colloid Interface Sci..

[bib35] Mariette F. (2018).

[bib36] Markley J.L., Horsley W.J., Klein M.P. (1971). Spin-lattice relaxation measurements in slowly relaxing complex spectra. J. Chem. Phys..

[bib37] McGee H. (2007).

[bib38] Meiboom S., Gill D. (1958). Modified spin-echo method for measuring nuclear relaxation times. Rev. Sci. Instrum..

[bib39] Métais A., Mariette F. (2003). Determination of water self-diffusion coefficient in complex food products by low field 1H PFG-NMR: Comparison between the standard spin-echo sequence and the T1-weighted spin-echo sequence. J. Magn. Reson..

[bib40] Odeblad E., Westin B. (1958). Proton magnetic resonance of human milk. Acta Radiol..

[bib41] Peemoeller H., Shenoy R., Pintar M. (1981). Two-dimensional NMR time evolution correlation spectroscopy in wet lysozyme. J. Magn. Reson..

[bib42] Schlesser J.E., Schmidt S.J., Speckman R. (1992). Characterization of chemical and physical changes in camembert cheese during ripening. J. Dairy Sci..

[bib43] Song Y.Q. (2009). A 2D NMR method to characterize granular structure of dairy products. Prog. Nucl. Magn. Reson. Spectrosc..

[bib44] Song Y.-Q., Venkataramanan L., Hürlimann M.D., Flaum M., Frulla P., Straley C. (2002). T1–T2 correlation spectra obtained using a fast two-dimensional Laplace inversion. J. Magn. Reson..

[bib45] Tanner J.E. (1970). Use of the stimulated echo in NMR diffusion studies. J. Chem. Phys..

[bib46] Telkki V.V. (2018). Hyperpolarized Laplace NMR. Magn. Reson. Chem..

[bib47] Tidona F., Alinovi M., Francolino S., Brusa G., Ghiglietti R., Locci F., Mucchetti G., Giraffa G. (2021). Partial substitution of 40 g/100 g fresh milk with reconstituted low heat skim milk powder in high-moisture mozzarella cheese production: Rheological and water-related properties. Lebensm. Wiss. Technol..

[bib48] Urbańczyk M., Bernin D., Koźmiński W., Kazimierczuk K. (2013). Iterative thresholding algorithm for multiexponential decay applied to PGSE NMR data. Anal. Chem..

[bib49] van den Enden J.C., Rossell J.B., Vermaas L.F., Waddington D. (1982). Determination of the solid fat content of hard confectionery butters. J. Am. Oil Chem. Soc..

[bib50] Venkataramanan L., Song Y.Q., Hürlimann M.D. (2002). Solving Fredholm integrals of the first kind with tensor product structure in 2 and 2.5 dimensions. IEEE Trans. Signal Process..

[bib51] Watanabe H., Fukuoka M. (1992). Measurement of moisture diffusion in foods using pulsed field gradient NMR. Trends Food Sci. Technol..

[bib52] Webb G.A., Belton P.S., Gil A.M., Delgadillo I. (2001).

[bib53] Wolter B., Krus M. (2006).

